# GH safety workshop position paper: a critical appraisal of recombinant human GH therapy in children and adults

**DOI:** 10.1530/EJE-15-0873

**Published:** 2015-02

**Authors:** D B Allen, P Backeljauw, M Bidlingmaier, B M K Biller, M Boguszewski, P Burman, G Butler, K Chihara, J Christiansen, S Cianfarani, P Clayton, D Clemmons, P Cohen, F Darendeliler, C Deal, D Dunger, E M Erfurth, J S Fuqua, A Grimberg, M Haymond, C Higham, K Ho, A R Hoffman, A Hokken-Koelega, G Johannsson, A Juul, J Kopchick, P Lee, M Pollak, S Radovick, L Robison, R Rosenfeld, R J Ross, L Savendahl, P Saenger, H Toft Sorensen, K Stochholm, C Strasburger, A Swerdlow, M Thorner

**Affiliations:** 1 University of Wisconsin School of Medicine and Public Health, Madison, Wisconsin, USA; 2 Cincinnati Children's Hospital Medical Center, Cincinnati, Ohio, USA; 3 Medizinische Klinik und Poliklinik IV, Klinikum der Universität München, Munich, Germany; 4 Massachusetts General Hospital, Boston, Massachusetts, UK; 5 Federal University of Parana, Curitiba, Brazil; 6 Department of Endocrinology, Skane University Hospital, Malmö, Sweden; 7 University College London Hospital and UCL Institute of Child Health, London, UK; 8 Hyogo Prefectural Kakogawa Medical Center, Hyogo, Japan; 9 Aarhus University Hospital, Aarhus, Denmark; 10 Molecular Endocrinology Unit, ‘Bambino Gesù’ Children's Hospital, Tor Vergata University, Rome, Italy; 11 University of Manchester and Manchester Academic Health Science Centre, Manchester, UK; 12 UNC School of Medicine, Chapel Hill, North Carolina, USA; 13 USC Leonard Davis School of Gerontology, University of Southern California, Los Angeles, California, USA; 14 Istanbul Faculty of Medicine, Istanbul, Turkey; 15 Centre Hospitalier Universitaire-Ste-Justine, Montreal, Quebec, Canada; 16 University of Cambridge, Cambridge, UK; 17 Indiana University School of Medicine, Indianapolis, Indiana, USA; 18 Perelman School of Medicine, Children's Hospital of Philadelphia, University of Pennsylvania, Philadelphia, Pennsylvania, USA; 19 Baylor College of Medicine, Houston, Texas, USA; 20 Christie Hospital NHS Foundation Trust, Manchester, UK; 21 Princess Alexandra Hospital, Brisbane, Queensland, Australia; 22 Stanford University School of Medicine, Stanford, California, Australia; 23 Erasmus University Medical Center, Rotterdam, The Netherlands; 24 Department of Endocrinology, Sahlgrenska Academy, Sahlgrenska University Hospital and Institute of Medicine, University of Gothenburg, Gothenburg, Sweden; 25 Department of Growth and Reproduction and EDMaRC, Rigshospitalet, København, Denmark; 26 Edison Biotechnology Institute, Ohio University, Athens, Ohio, USA; 27 Penn State College of Medicine, Hershey Medical Center, Hershey, Pennsylvania, USA; 28 McGill University, London, UK; 29 Johns Hopkins University School of Medicine, Baltimore, Maryland, USA; 30 St. Jude Children's Research Hospital, Memphis, UK; 31 Oregon Health and Science University, Portland, Oregon, USA; 32 University of Sheffield, Sheffield, UK; 33 University of Sheffield, Sheffield, UK; 34 Winthrop University Hospital, SUNY Stony Brook, Mineola, New York, USA; 35 Aarhus University Hospital, 8000, Aarhus, Denmark; 36 Charité Universitätsmedizin Berlin, Berlin, Germany; 37 Institute of Cancer Research, University of London, London, UK; 38 Emeritus University of Virginia, Charlottesville, Virginia, USA

## Abstract

Recombinant human GH (rhGH) has been in use for 30 years, and over that time its safety and efficacy in children and adults has been subject to considerable scrutiny. In 2001, a statement from the GH Research Society (GRS) concluded that ‘for approved indications, GH is safe’; however, the statement highlighted a number of areas for on-going surveillance of long-term safety, including cancer risk, impact on glucose homeostasis, and use of high dose pharmacological rhGH treatment. Over the intervening years, there have been a number of publications addressing the safety of rhGH with regard to mortality, cancer and cardiovascular risk, and the need for long-term surveillance of the increasing number of adults who were treated with rhGH in childhood. Against this backdrop of interest in safety, the European Society of Paediatric Endocrinology (ESPE), the GRS, and the Pediatric Endocrine Society (PES) convened a meeting to reappraise the safety of rhGH. The ouput of the meeting is a concise position statement.

## Introduction

Recombinant human growth hormone (rhGH) has been in use for 30 years, and over that time its safety and efficacy in children and adults has been subject to considerable scrutiny. Prior to 1985, cadaveric pituitary-derived GH was used, but this was stopped in most countries that year, following the recognition that it could transmit Creutzfeldt–Jakob disease, with patients still being diagnosed after incubation periods upto 40 years (1). In 2001, a statement from the GH Research Society (GRS) concluded that ‘for approved indications, rhGH is safe’ (2); however, the statement highlighted a number of areas for on-going surveillance of long-term safety, including cancer risk, impact on glucose homeostasis, and use of high dose pharmacological rhGH treatment. Over the intervening years, there have been a number of publications addressing the safety of rhGH with regard to mortality, cancer and cardiovascular risk, and the need for long-term surveillance of the increasing number of adults who were treated with rhGH in childhood.

Against this backdrop of interest in safety, the European Society of Paediatric Endocrinology (ESPE), the GRS, and the Pediatric Endocrine Society (PES) convened a meeting to reappraise the safety of rhGH. Invitees included pediatric and adult endocrinologists, epidemiologists, and medical/safety representatives from the pharmaceutical industry, with the latter being asked to share data regarding safety from their own databases. Review papers (including meta-analyses of safety data) were written in preparation for the meeting (3, 4), and all participants were provided in advance with the key literature, which formed the basis for the group discussions (see Supplemental References, see section on supplementary data given at the end of this article). The quality of the evidence from the literature was not formally graded as there are no randomized controlled trials on safety issues and a significant amount of the safety literature during GH treatment years is derived from post marketing surveillance studies. A planning committee of academic pediatric and adult endocrinologists chose the topics, designed the program, and formulated the questions for discussion in break-out groups (Supplemental Data: Meeting Program).

Break-out group reports were discussed in plenary sessions aimed at generating the majority view on the responses to the questions posed at the workshop. Writing groups at the end of days 1 and 2 of the meeting compiled their reports, and these were brought together into a final statement that was shared with and revised by participants on the last day, edited further, and sent for final review after the meeting. When no agreement was reached on specific points, a majority vote was taken; when there was no majority, there was further discussion, rewording of the document, and the vote was repeated until a majority opinion was obtained. Representatives from medical/safety departments of pharmaceutical companies participated in the discussions and presented data during the first 2 days. They were not present during the writing and voting process on the last day. However, they were asked to review the manuscript for factual errors about their data after completion of the final draft.

The text of this statement was based on: i) the combined comments of the break-out groups to the questions, ii) the combined comments of the whole group during plenary discussions, iii) knowledge of the current literature, and iv) the combined experience of clinicians and scientists active in the GH field. This report is not intended to be an exhaustive review of the literature, but is a concise report of the proceedings of the workshop.

## Mortality and cancer risk

In evaluating the available evidence addressing mortality and cancer risk among GH-treated populations, critical assessment of the strengths and limitations of published research and safety data presented within the meeting was undertaken. Features related to limitations in the quality, and thus interpretation of these data, are highlighted below.

### Overall mortality and GH treatment

The group recognized that many of the disorders treated with GH in children and adults have an inherently higher mortality risk, which is related to the underlying disorder. In most studies therefore the potential impact of GH treatment on overall mortality is difficult to distinguish from the impact of the underlying disorder. This was also the conclusion reached on neoplasia risk in GH-treated children published after the workshop (5).

The agreement reached was that aggregate evidence from available datasets does not support an association between ongoing or previous GH therapy and all-cause mortality. The group acknowledged that some individual reports and meta-analyses had indicated association (3). However, major concern was raised about lack of appropriate comparison cohorts of untreated patients, incomplete data on GH exposure and treatment regimens, inadequate characterization of patients, and inadequate identification of other risk factors. In particular, problems with duration and completeness of follow-up, study designs used to date, and reported risk metrics make it difficult to reach definitive conclusions about a causal relationship between GH treatment and all-cause mortality. It is also necessary to distinguish relative risk and the standardized mortality ratio from other metrics such as absolute risk and number-needed-to-harm, with the latter being more relevant to counseling patients and families about risk (Table 1).

The ongoing multicenter ‘Safety and Appropriateness of Growth hormone treatments in Europe’ (SAGhE) study, which is assessing mortality in adults previously treated with rhGH in childhood for approved indications, will add further information to this field. A full description of the cohort and the methodologies that are being used to assess mortality and cancer incidence risks is provided in Swerdlow *et al*. (published after the workshop was held) (6).

### Cause specific mortality and GH treatment

The group agreed that available data are inadequate for reaching conclusions regarding any influence of GH on cause-specific mortality. As noted above for all-cause mortality, the key question in assessing the potential impact of GH treatment on subsequent mortality concerns the contribution of GH deficiency itself to mortality risk. It is generally accepted that adult GH deficiency and certain short-stature syndromes are associated with elevated mortality in the untreated state. However, cause-specific mortality rates for persons with these disorders who are not treated with GH have not been quantified precisely enough to allow comparison with GH-treated patients.

### Risk of new primary cancers

Available data in children do not indicate an increased risk of new primary cancers in GH recipients (Table 2). The data for new cancer risk in adult GH recipients are reassuring (Table 2). However, there are limitations to all these statements. A variety of information sources are available in relation to cancer risk among GH-treated patients, including post-marketing surveillance (phase 4) studies, a limited number of other cohort studies, and clinical series. While some of these data sources are large and include many patient years of observation with generally reassuring results, the number of subjects with long duration of follow-up is small and data are incomplete, precluding definitive long-term safety conclusions. Other weaknesses are insufficient control for selection bias, inadequate sample sizes to assess cancers with low incidence, and lack of appropriate comparison populations.

The group did not support cancer surveillance beyond local standard practice in patients (children and adults) currently treated with GH nor in those previously treated with GH (including those with a previous malignancy).

### Risk of recurrence of a previous primary cancer

Available data in children do not indicate an increased risk of recurrence of primary cancer in GH recipients (Table 2). The data in adult GH recipients are presently insufficient to address this situation, but available data on benign pituitary tumors do not indicate an increased risk of recurrence during long-term GH replacement (4) (Table 2).

### Risk of second or subsequent neoplasms

The risk of second primary tumors in GH-treated survivors of pediatric cancers was reported to be elevated in one study population, with highest risk early after GH treatment and declining with longer follow-up (7, 8, 9). The general opinion was that the association between GH therapy and risk of second tumors is insufficient to preclude use of rhGH for licensed indications in children (Table 2). Data are insufficient in patients surviving adult-onset malignancies to reach a conclusion about safety of GH use in this population (Table 2). It is recommended that potential risk be discussed with patients and their families.

### Initiation of GH therapy after cancer treatment

Few data are available to provide guidance on the appropriate interval between completion of cancer therapy and initiation of GH treatment in both children and adults. Therefore in deciding on this interval, consideration should be given to factors related to the tumor, time elapsed since completion of cancer treatment, and the importance of initiating GH treatment in the individual patient (e.g. severity of growth failure if not treated with GH).

### Use of GH therapy in patients with a background risk for cancer

Definitive data are lacking regarding the safety of GH therapy in ‘high risk’ patients (in particular children), including those with syndromes, diseases, and mutations known to be associated with an inherent elevated risk for cancer and early mortality (e.g. Neurofibromatosis type 1, Fanconi anaemia, or Down syndrome). Therefore, the decision to start GH therapy should be carefully considered and discussed with families.

## Stroke, cardiovascular disease, and metabolic risk

### Stroke

There was agreement that data were inadequate to determine whether GH therapy in childhood increases risk of stroke in young adults. The rationale for reaching this conclusion was that in the one published study reporting an association (10), the number of subjects was small and the risk of developing this complication in a comparable population was unknown. This single study reported 11 validated cases of stroke, including subarachnoid hemorrhage, intracerebral hemorrhage, and ischemic stroke at a mean age of 24±7 years, out of a population of 6874 patients with either isolated idiopathic GH deficiency or short stature in those born small for gestational age (SGA), or idiopathic short stature (ISS), who started treatment with rhGH between 1985 and 1996. Absolute risk of stroke was still small in this population (1.6/1000 persons) and there may have been potential confounding factors; data were lacking on family history, concomitant medications, smoking, or hypertension. Stroke is a potential serious complication that warrants further scrutiny, but at present the evidence is insufficient to raise stroke as a concern with families before starting GH treatment in children.

### Cardiovascular disease and metabolic risk

Multiple studies have analyzed the effects of GH therapy on risk factors for cardiovascular and metabolic disease. Administration of GH modulates insulin sensitivity in a complex manner influenced by numerous factors such as age, body composition, and duration of therapy. The incidence of developing glucose intolerance or overt type 2 diabetes mellitus (T2D) during GH treatment in pediatric patients with GH deficiency or ISS is very low (11). Although the lifetime risk of glucose intolerance and T2D in a number of conditions treated with GH, including Turner syndrome (TS) and in short children born SGA, is higher than in the background population, GH treatment does not increase the incidence of T2D in these conditions in the short term.

In a subset of adult GHD patients with a propensity toward development of T2D, such as obesity and/or family history of T2D, GH therapy can be associated with the development of glucose intolerance or T2D in the first year of therapy (4), so monitoring with HbA1c is important.

GH reduces visceral fat and leads to an increase in lean body mass. Cardiovascular risk markers are increased in children and adults with GHD; these can improve with administration of GH. GH has also been shown to reduce LDL cholesterol, and there is a suggestion that GH can increase HDL cholesterol and reduce carotid intimal thickness; however, it has not been clearly demonstrated that GH replacement decreases the rate of cardiovascular events (4).

There is no increase in blood pressure (BP) in children or adults on GH therapy. In fact there is a modest reduction in diastolic BP with GH administration in short SGA children and in adults with GHD.

## Managing recognized side-effects

The side-effects described below may be related to the use of GH. They can occur independent of GH and the role of GH should be considered on an individual basis.

### Intracranial hypertension

Intracranial hypertension (ICH) may occur secondary to GH therapy in children, but can be difficult to confirm. The absence of papilledema does not exclude the diagnosis. Symptoms resolve with discontinuation of GH, which then can be restarted at a lower dose and gradually increased. Persistent severe headaches that do not resolve with discontinuation of GH therapy should be further evaluated by a neurologist.

### Musculoskeletal symptoms

In adults, edema, carpal tunnel syndrome, and musculoskeletal aches and pains related to fluid retention may be a sign of GH over-dosage. These symptoms rarely occur in adults if GH is started at a low dose and titrated up. In children, musculoskeletal aches may be related to increased growth velocity or underlying conditions rather than the GH treatment.

### Scoliosis

Scoliosis is more prevalent in patients with TS or Prader–Willi syndrome (PWS) even in the absence of GH treatment. Progression of scoliosis can be accelerated by rapid growth, such as the pubertal growth spurt, and is not associated with GH treatment *per se*. Clinical examination of the spine should be occur before start of therapy and at follow-up of pediatric patients receiving GH therapy. Even in the presence of scoliosis, GH therapy can be initiated or continued, though radiographic studies should be obtained to monitor for any change.

### Slipped capital femoral epiphysis

Slipped capital femoral epiphysis has been associated with GH therapy in children and is likely due both to growth acceleration and the underlying condition. Radiographic studies and appropriate referral are urgently required.

### Obstructive sleep apnea

GH can stimulate adenotonsillar growth and may thereby exacerbate obstructive sleep apnea, particularly in patients receiving GH treatment for PWS. Polysomnography prior to initiating therapy and monitoring during GH treatment is recommended for patients with PWS (12). Occurence of obstructive sleep apnea is also increased in obese adults with GHD and if exacerbated with GH treatment, a dose reduction should be considered.

### Pancreatitis

Pancreatitis has been observed to occur in children receiving GH therapy. While this side-effect is listed on the package insert, it is extremely rare and its causal relationship to GH treatment remains unclear. However, if a child on GH therapy develops severe abdominal pain, pancreatitis should be considered.

### Alterations in cortisol and thyroid metabolism with GH treatment

GH increases the tissue conversion of active cortisol to inactive cortisone. Thus, initiation of GH therapy in patients with subclinical adrenocorticotropic hormone deficiency may induce symptomatic adrenal insufficiency requiring glucocorticoid substitution, and patients already on cortisol replacement may need dose adjustment.

GH increases the peripheral conversion of thyroxine (T_4_) to tri-iodothyronine. Commencement of GH replacement may therefore unmask pre-existing central hypothyroidism as defined by a fall of serum-free T_4_ into the subnormal range. In patients with hypopituitarism already taking T_4_, adjustment of the T_4_ dose may be needed after initiation of GH replacement therapy if a decrease in the serum concentration of free T_4_ occurs. Thyroid function should therefore be monitored at initiation of GH treatment and after dose increases.

For children with isolated, idiopathic GHD without hypothyroidism and no abnormality on magnetic resonance imaging (MRI) of the hypothalamic–pituitary region, routine evaluation of adrenal function is not required unless symptoms develop. For patients of any age with reason for concern regarding evolving pituitary hormone deficiencies, such as following irradiation or mutations associated with evolving hypopituitarism, the hypothalamic–pituitary–adrenal and –thyroid axes should be evaluated regularly.

## Dosing and monitoring of GH therapy

### Risk reduction: pediatric patients

Baseline clinical evaluation should be conducted based on the condition prompting GH treatment. For example, full pituitary function testing and MRI of the brain, with special attention to the hypothalamic–pituitary region, are indicated in children with GH deficiency. The primary objective of GH therapy in children is to obtain a satisfactory growth response without incurring adverse events.

Measurement of insulin-like growth factor 1 (IGF1) concentration is recommended for children with GH deficiency treated with GH, with the goal of normalizing serum IGF1 concentrations. However, the group recognized the lack of evidence base supporting the value of IGF1 monitoring for safety in children and the lack of any data to indicate a safe upper limit for serum IGF1 concentrations. Some epidemiological studies in healthy adults without GHD have suggested associations between serum IGF1 concentrations in the upper part of the normal range or above and some forms of cancer, and between serum IGF1 concentrations in the lower part of the normal range and cardiovascular disease. The relevance of such studies for pediatric patients or GHD adults treated with GH has not been established.

When the growth response is not satisfactory in children with TS, short children born with SGA, and those with chronic renal insufficiency, the GH dose can be increased within the recommended range in order to achieve the desired growth response. This can lead to IGF1 concentrations above the normal range (>+2 SDS). There is no clinical evidence at this time in children that raising the IGF1 into this higher range for a period of time carries an increased risk of adverse events. However, this situation has not been rigorously evaluated and aiming for IGF1 levels in the normal range is recommended.

Monitoring of GH treatment should include: bone age assessment, thyroid function testing (in GH-deficient patients), and as indicated above, adrenal function testing in patients with evidence or suspicion of multiple pituitary hormone deficiency (e.g. transcription factor defects, midline abnormalities). Additional general safety monitoring for non-GH deficient patients should include clinical assessment for scoliosis and monitoring of HbA1c levels (for further details in specific conditions refer to published reports) (12, 13, 14, 15).

Following completion of linear growth, GH deficiency should be re-evaluated during the transition period (16, 17). When ongoing GH deficiency is diagnosed, we recommend referral to an adult endocrinologist for consideration of adult GH replacement.

### Risk reduction: adult patients

Adult patients with GH deficiency should provide a detailed medical history, with particular attention to past or present history and family history of malignancy and DM, followed by physical examination, pituitary function testing, and pituitary MRI. Repeat imaging may not be required in childhood onset GH-deficient patients. GH replacement is generally initiated with a low dose of GH and then titrated up based on serum IGF1 concentrations and monitoring for fluid retention related symptoms. The goal is to achieve hormone replacement with normal age-adjusted serum IGF1 values. Safety monitoring includes measurement of HbA1c and in selected patients, additional hormonal testing as discussed above. Oral estrogen reduces the sensitivity to GH and therefore any change in oral estrogen use should prompt IGF1 measurement and re-evaluation of the GH dose.

rhGH is continued in some countries during pregnancy until about 20 weeks of gestation, after which time placental GH concentrations are sufficient to maintain normal serum IGF1 concentrations. In other countries, GH treatment is discontinued when a patient becomes pregnant.

### GH treatment during critical illness

GH treatment used at high doses in critically ill patients is associated with increased mortality (18). There are however no data on the impact of GH replacement in GHD patients during critical illness. The group agreed that GH-deficient children and adults hospitalized with a critical illness should only be administered GH in physiologic doses. GH treatment should be stopped in critically ill patients treated with GH for non-GH deficient indications because these patients may be receiving supraphysiological GH doses while retaining endogenous GH secretion.

## Off-label use of GH

The off-label use of GH therapy outside of approved indications is not endorsed, but it is recognized that GH is being prescribed for growth promotion in a variety of conditions with differences between countries. For example, the use of GH for children with ISS, Noonan syndrome, *SHOX*-gene haplo insufficiency, and chronic renal insufficiency are indications that have been approved for GH treatment in children in some countries. For certain other conditions, there is some evidence for a beneficial effect of GH therapy. These include cystic fibrosis (CF), inflammatory disorders (e.g. inflammatory bowel disease, juvenile idiopathic arthritis), and mild forms of skeletal dysplasia (e.g. hypochondrodysplasia). The benefit as well as safety profile in these indications has not been substantiated, and for certain disorders may involve increased risks (e.g. glucose intolerance in CF, ICH in hypochondroplasia).

In other situations, GH is prescribed to treat conditions despite lack of substantial scientific evidence for either efficacy or safety. The group agreed that GH should not be used ‘off-label’ except in clinical trials that can assess efficacy and safety or on an approved compassionate indication protocol. In some countries, it is illegal to prescribe GH for off-label indications.

### Abuse of GH

There was full agreement that rhGH should not be administered for performance enhancement, anti-aging, or other illicit uses. Toxicity from such interventions is usually not reported, raising the concern that serious adverse reactions may occur especially when GH is combined with other supplements such as anabolic steroids.

## Conclusions

Fourteen years after the 2001 GRS consensus statement (2), the safety record of rhGH remains good, supported by evidence from the follow-up of thousands of children and adults over tens of thousands of patient years. Available information on the safety of GH is derived from a wide-range of sources from around the world, which have been of significant value in formulating the current statement (Supplemental References). Out of necessity, safety-related issues in GH-treated populations must rely on results from observational research which has a greater number of methodological limitations than randomized placebo-controlled studies (Table 3), with the latter considered unethical for a therapy that has proven benefit. After detailed consideration of such limitations, the group concluded that GH continues to have a good safety record when used for approved indications and at recommended doses.

Nevertheless, the group agreed that continued surveillance of those exposed to rhGH is essential both during and in the years after treatment and into old age in those who continue therapy. This is particularly important with the advent of long-acting GH preparations with very different pharmaco-kinetic and -dynamic profiles compared to daily rhGH injections.

For future consideration, the group proposed the establishment of a carefully designed and rigorously conducted cohort study to provide a single research resource for testing hypotheses that specifically address the long-term safety, vascular and metabolic health, psychosocial, and quality of life outcomes of GH treated patients. Beyond formal hypothesis testing, a large cohort would provide opportunities to collect new data from GH-exposed patients. Any new initiative would need to incorporate: i) appropriate comparison population(s) to permit more direct assessment of outcomes between GH-exposed and non-treated groups; ii) mechanisms for direct patient contact for longitudinal reporting of health-related and quality of life outcomes; and iii) ability to validate selected self-reported outcomes. A comprehensive cohort study has the potential to contribute substantially to the knowledge base that informs the clinical care and long-term management of patients who have received GH treatment.

## Supplementary data

This is linked to the online version of the paper at http://dx.doi.org/10.1530/EJE-15-0873.

## Figures and Tables

**Table 1:** Terms and their definitions used to quantify risk.


Relative risk is based on a comparison of the risk in GH treated patients, or a subset of such patients, vs the risk in an untreated comparison group or population
The standardized mortality ratio (SMR) is the ratio of observed cases among GH treated patients, or a subset of such patients, to the expected number of cases based upon the general population rate
The absolute risk is the calculated rate, generally expressed as number of cases per 1000, 10 000, or 100 000 person years
The number-needed-to-harm is defined as the number of patients needed to be exposed to a risk factor over a specified period to cause harm in one patient

**Table 2:** Majority view of the effect of GH treatment for approved indications on cancer risk in children and adults (including those with a childhood-onset of GH deficiency). The term ‘robust’ is used when there are multiple independent published sources supporting the statement (see Supplemental References). The term ‘suggestive’ is used when there are less than three sources supporting the statement. The term ‘insufficient’ is used when available publications provide inadequate evidence to support the statement.

**Age at onset of GH treatment**	**New primary cancer**	**Recurrence of the primary cancer in survivors**	**Second or subsequent neoplasm in survivors**
Child	No evidence for GH treatment effect Level: robust	No evidence for GH treatment effect Level: robust	Risk present but diminishes with time from onset of GH treatment Level: suggestive
Adult	No evidence for GH treatment effect Level: suggestive	Insufficient data available	Insufficient data available

**Table 3:** Data limitations related to safety issues.


Insufficient duration and unknown completeness of follow-up
Lack of appropriate comparison populations
Lack of complete documentation of GH dose-specific exposure
Lack of dose-specific assessment of IGF1 concentrations
Insufficient control for selection bias
Inconsistent definition and validation of outcomes
Insufficient sample sizes to allow assessment of low incidence outcomes
Reporting bias and lack of sensitivity to detect more subtle effects
